# Adolescent body weight and health-related quality of life rated by adolescents and parents: the issue of measurement bias

**DOI:** 10.1186/s12889-015-2533-4

**Published:** 2015-11-30

**Authors:** Pranav K. Gandhi, Dennis A. Revicki, I-Chan Huang

**Affiliations:** School of Pharmacy, South College, 400 Goodys Ln, Knoxville, TN 37922 USA; Outcomes Research, Evidera, 7101 Wisconsin Ave., Suite 1400, Bethesda, MD 20814 USA; Department of Epidemiology and Cancer Control, St. Jude Children’s Research Hospital, 262 Danny Thomas Place, Memphis, TN 38105 USA

**Keywords:** Adolescents, Differential item functioning, Health-related quality of life, Obesity, Measurement bias

## Abstract

**Background:**

Evidence is sparse about whether body weight categories in adolescents are associated with differences in pediatric HRQoL rated by adolescents and parents. Additionally, it is unknown whether HRQoL rated by individuals with different body mass index (BMI) weight categories is psychometrically comparable. This study aimed to assess whether difference in pediatric HRQoL rated by adolescents and their parents was explained by BMI weight status, and to test measurement properties of HRQoL items related to weight categories using differential item functioning (DIF) methodology. DIF refers to the situation when the individuals across subgroups rate an item differently (e.g., item score three by one subgroup and four by another) given the same underlying construct.

**Methods:**

A cross-sectional study utilizing a sample of parents (*n* = 323) and their adolescents aged 15–18 years old (*n* = 323) who enrolled in Florida’s Medicaid. Adolescent self-reports and parent proxy-reports of the Pediatric Quality of Life Inventory was adopted to measure pediatric HRQoL. We classified body weight categories as normal weight, overweight, and obesity. A Multiple Indicator Multiple Cause (MIMIC) method was used to assess DIF associated with BMI weight status, especially testing the disparity in the parameters of different weight categories (reference: lower weight category) associated with a response to a HRQoL item conditioning on the same underlying HRQoL. DIF analyses were conducted by adolescent self-reports and parent proxy-reports.

**Results:**

Parents reported lower pediatric HRQoL across all domains than adolescents did. Excess body weight (combined overweight and obese) was significantly associated with a greater discrepancy in the rating of emotional and total functioning between adolescents and parents (*p* < 0.05). DIF associated with BMI weight categories was identified by two items in adolescent self-reports and five items in parent proxy-reports.

**Conclusions:**

Adolescents’ BMI weight categories significantly contribute to a difference in the rating of pediatric HRQoL by adolescents and parents.

## Background

Obesity is a significant cause of disability and lower life expectancy [1]. The estimated prevalence of obesity among adolescents in the United States between 2011 and 2012 was 20.5 % [2]. Compared to normal weight peers, obese children and adolescents are more likely to develop several chronic diseases which usually occur later in life [3, 4]. Obese children and adolescents are often stigmatized and discriminated against in society, resulting in increased loneliness, sadness, and social isolation [5].

Several studies have investigated the relationship between obesity and health-related quality of life (HRQoL) in children and adolescents [6–10]. Evidence suggests that obese children and adolescents reported poorer overall HRQoL compared to their lean counterparts [6–10]. A systematic review found a negative association between body mass index (BMI) and HRQoL measured by the Pediatric Quality of Life Inventory (PedsQL) [7]. When comparing obese and overweight children to their lean counterparts, a moderate to strong impairment was found in physical, social, and emotional functioning, while a minimal impairment was found in school functioning [7]. Health outcomes (e.g., HRQoL) related to obesity in adolescents of low-income/minority families deserve our attention because the prevalence of overweight and obese status was more prevalent in this vulnerable group than those of high-income/non-minority families [2].

The use of parent proxy-reports for a child’s health outcomes is recommended if children are mentally disabled, too young, or too sick to self-report [11]. Literature consistently demonstrates that parents’ observations of their children’s HRQoL tend to be lower than that of children’s self-reports [4, 7–12]. Additionally, both children and parents reported lower physical and social functioning scores for obese children compared to normal weight children [9]. The difference in pediatric HRQoL rated by children/adolescents and parents was determined by age, gender, social economic status, and health status [9, 13], and the difference increased with the child’s age [7, 9]. Adolescence is an important stage of human development. In this stage unhealthy lifestyle and behaviors are frequently introduced to adolescents, which increase the likelihood of overweight and obesity [2]. However, little is known about whether the adolescent’s BMI is associated with the difference in pediatric HRQoL ratings by adolescents themselves and their parents. Evaluating the contribution of BMI to the discrepancy in pediatric HRQoL rated by adolescents and parents improves our understanding about how body weights bias the perceptions of HRQoL by both stakeholders.

Assessing HRQoL is a subjective task that involves a complex cognitive process which is influenced by the subject’s bio-psycho-social factors, such as personality and health status [14]. It is likely that people with long-term chronic diseases may change their internal standards to conceptualize, perceive, and rate HRQoL compared to healthy people [15]. It is important to investigate the equality in the response of a specific item of HRQoL between different groups of participants given the same underlying HRQoL (e.g., emotional functioning). Underlying HRQoL (or latent trait of HRQoL) is a psychometric terminology frequently used to describe the notion that HRQoL is unobservable concept, but can be measured by assessment tools comprised of content-appropriate items. Without establishing measurement equivalence, assessments of HRQoL ratings across groups of participants may not be comparable, leading to invalid or biased finding. It is unclear whether HRQoL rated by individuals with different BMI weight categories are equivalent and comparable, especially in the adolescent population. The issue of stigma and discrimination associated with being overweight or obese may influence obese children (and their parents) to rate the items of HRQoL differently compared to normal weight children (and their parents) [16, 17].

Differential item functioning (DIF) analysis is a psychometric method and frequently used to investigate the equality in the response of a specific item between different groups of subjects after matching them on the same underlying construct of HRQoL [14, 18, 19]. Theoretically, if the underlying HRQoL (e.g., emotional functioning) is the same between an obese adolescent and his/her lean counterpart, we would expect that both adolescents will have the same probability of responding to a particular category of an item measuring the underlying HRQoL. However, if different groups of individuals (e.g., obese and lean adolescents) respond differently to the items given the same level of HRQoL, it can potentially lead to serious threats to the measurement validity. A DIF phenomenon exists when this equality assumption does not hold, leading to an over- or under-estimated HRQoL score of an adolescent. If DIF of an item related to the group membership is identified (e.g., obese and lean adolescents), it implies this item may be interpreted or perceived differently by adolescents between two groups, which may threaten the validity of HRQoL measures, especially when comparing HRQoL between obese and lean adolescents. Multiple-Indicator Multiple-Cause modeling (MIMIC) [20] is one of DIF methods that help analyze item response function and group difference. To the best of our knowledge, the MIMIC method has never been used to assess DIF in HRQoL measures associated with different BMI weight categories.

Our first objective of this study was to assess whether there was a difference in pediatric HRQoL rated by adolescents and parents of low-income families. In particular, we examined the extent to which BMI weight categories in adolescents and socio demographic factors contributed to the differences in pediatric HRQoL rated by adolescents and parents. Our second objective was to examine whether there was a difference in pediatric HRQoL rated by adolescents and parents across different BMI weight categories. In particular, we examined whether the difference in pediatric HRQoL across different BMI weight categories in adolescents was explained by DIF associated with BMI using the MIMIC methodology. Dyadic data collected from adolescents and parents who enrolled in Florida’s Medicaid Program were used for analyses.

## Methods

### Study sample and data sources

This is a cross-sectional study with data collected from a previous study [19] comprised of parents and their adolescents enrolled in Florida’s Children’s Medical Services Network program (KidCare) in 2005. KidCare is a public insurance program that provides coverage for children who are uninsured under the age of 19 years and whose family has incomes up to 200 % of the federal poverty level. All adolescents in this sample were also enrolled in Medicaid. In Florida, Medicaid is a medical assistance program that is managed by the Agency for Health Care Administration to provide health care services to low-income individuals and families [21]. University of Florida’s Institutional Review Board (IRB) approved the study protocol. Per University’s IRB, we obtained a waiver of collecting written informed consent by collecting verbal agreement from all study participants over the phone when we enrolled them.

We identified a statewide random sample comprised of 700 adolescents from the enrollment files maintained by the Florida Children’s Medical Services Network. The use of this sampling frame was on the basis of sample size needed (at least 230 dyads of adolescents and parents) for psychometric analyses in our previous study [19], which is also appropriate for the current study. A telephone survey using a structured questionnaire was conducted for families with an adolescent 15 through 18 years old living with them between 12/2005 and 03/2006. Multiple callbacks (at a maximum of 10 times) were performed if phone numbers were busy or not answered. Eleven percent of the families had disconnected phone numbers or did not answer the calls; 25 % of parents reported that their adolescents were physically or mentally unable to complete the survey; 6 % refused to allow their adolescents to be interviewed; and 4 % of the parents subsequently refused to participate after initially granting permission. As a result, the study sample consisted of 376 dyads of adolescents and their parents who completed the survey (survey response rate: 54 %). Thirty-seven dyads were excluded from the final statistical analyses since they had more than 50 % of items missing in HRQoL survey [19].

### BMI categorization

The adolescent’s weight and height were self-reported by the parent. BMI was calculated as the weight in kilograms divided by the height in meters squared. Age-and-sex growth charts developed by the U.S. Centers for Disease Control and Prevention [22] were used to categorize each adolescent into one of the following categories: obese (BMI ≥95th percentile), overweight (BMI ≥85th and <95th percentile), normal weight (BMI ≥5th and <85th percentile), and underweight (BMI <5th percentile). We excluded 16 adolescents with underweight since the mechanisms by which excess body weight (overweight and obese) and underweight that influence adolescents’ health and HRQoL might be different, leaving 323 dyads for the final analyses [3]. People who are underweight may experience poor HRQoL. The possible mechanisms through which underweight affects HRQoL are malnutrition or poor health conditions (e.g., depression and cancer) [3].

### PedsQL Core 4.0 for HRQoL measure

PedsQL Core 4.0 is a widely used validated generic instrument for pediatric HRQoL assessment [23–25]. We used both adolescent self-reports and parent proxy-reports to measure pediatric HRQoL. The PedsQL is comprised of 23 items covering four domains: physical (eight items) (e.g., “In the past month … It is hard for me to walk more than one block”), emotional (five items) (e.g., “In the past month … I feel afraid or scared”), social (five items) (e.g., In the past month … I have trouble getting along with other kids”), and school functioning (five items) (e.g., “In the past month … It is hard to pay attention in class”). A five-point response category for each item is utilized (from 0 = “never a problem” to 4 = “almost always a problem”). The specific domain score is calculated as the sum of the item responses divided by the number of items answered and scores are transformed which range from 0 to 100. The total HRQoL score is computed as the sum of all item responses divided by the number of items answered on all the domains. Higher item and domain scores indicate better HRQoL [23–25]. In our study sample, reliability coefficients (Cronbach’s alpha) of the adolescent self-reports were 0.82, 0.77, 0.76, 0.65, and 0.88 for the domains of physical, emotional, social, school, and total HRQoL, respectively. Reliability coefficients of the parent proxy-reports were 0.87, 0.79, 0.79, 0.73, and 0.91 for the domains of physical, emotional, social, school, and total HRQoL, respectively.

### Statistical analysis

Intra-class correlation coefficient (ICC) was estimated to demonstrate the magnitude of the agreement between pediatric HRQoL rated by adolescents and parents. Given that the domain scores of HRQoL were not normally distributed, we conducted Wilcoxon signed rank tests to investigate the differences in pediatric HRQoL rated by adolescents and parents by individual BMI weight categories. The Cohen’s effect size was calculated to quantify the magnitude of the difference and a two-tailed *p* < 0.05 was deemed the statistical significance. Effect sizes of <0.2, 0.2–0.49, 0.5–0.79, and ≥0.8 indicate “negligible’, ‘small’, ‘medium’, and ‘large’ differences, respectively [26]. We also performed a multiple regression analysis with robust standard errors to examine whether BMI weight categories in adolescents and other factors (such as the adolescent’s age, gender, and parent’s race and educational background) were associated with the differences in pediatric HRQoL rated by adolescents and parents. The selection of covariates was based on evidence from literature and results of our bivariate analyses [7–10]. In the regression analysis, normal weight was treated as a reference category compared to the overweight and obese categories.

We conducted Mann–Whitney U-tests to investigate the differences in pediatric HRQoL between different BMI weight categories. Mann–Whitney U-tests were conducted to compare self-reports (and parent proxy-reports) of pediatric HRQoL for obese adolescents versus normal weight adolescents, obese adolescents versus overweight adolescents, overweight adolescents versus normal weight adolescents, and normal weight adolescents versus excess body weight (defined as a combination of overweight and obese). The effect size was calculated to quantify the magnitude of the difference and a two-tailed *p* < 0.05 was used to determine the statistical significance.

### DIF methodology

DIF occurs when the individuals from subgroups (e.g., different BMI weight categories) rate an item unequally given the same underlying HRQoL (e.g., emotional functioning) the item intends to measure. Evidence of DIF in HRQoL items suggests the problematic construct validity of HRQoL measures because DIF implies misinterpreting the meaning of a HRQoL item between subgroups. In this study, we used MIMIC method to identify DIF associated with BMI weight categories in adolescents by incorporating additional background variables (e.g., the adolescent’s age, gender and parents’ race and educational background) into the analysis. The MIMIC model is a special case of structural equation model (SEM) and comprises two parts: a measurement model which defines the relations between a latent variable (a specific HRQoL domain) and its indicators (items measuring a specific HRQoL domain) and a structural model which specifies the relationships among latent variables and BMI weight status. Ideally, the relationships of BMI weight status with individual items of a specific HRQoL domain are explained through the relationship with a specific HRQoL domain. However, if the relationships of BMI weight status with individual HRQoL items exist, it will indicate presence of DIF. The technical merit of MIMIC methodology is the use of SEM framework to test a disparity in the magnitude of parameter for a group variable (e.g., over weight vs. normal weight) associated with a response to an item of emotional domain conditioning on the same underlying HRQoL (e.g., emotional functioning). In SEM, the underlying HRQoL (e.g., emotional functioning) is estimated through specific items (i.e., emotional items) that measure this specific HRQoL by incorporating measurement errors embedded in the items (i.e., variance of emotional items not estimated by the underlying emotional functioning). When the DIF issue was adjusted in the analysis, the comparison of HRQoL between different groups of individuals is regarded as unbiased and reflects the true difference.

In this study, serial tests of nested models, beginning with the most constrained model, sequentially relaxing cross-group equality constraints on the item parameters, and ending up with the least constrained model, were performed to detect DIF [27]. The procedures are iterative and inclusive of the following steps:

- Step 1: constraining the relationship between body weight (e.g., overweight and obese) and individual items of the PedsQL to be zero, and examining the modification indices to suggest how much the model fit would be improved if specific relationships were freely estimated, and
- Step 2: starting with an item with the largest and significant modification index, and adding individual items of the PedsQL one at a time to the model for freely estimating its relationship with body weight (e.g., overweight and obese) until no modification indices were greater than 3.84 (d.f. = 1).

We performed the DIF analyses using Mplus 6.0 [28], and conducted the rest of the analyses using SAS 9.1 [29].

## Results

### Characteristics of the study sample

Table 1 shows the characteristics of the 323 dyads of adolescents and parents who were included in the analyses. Of the 323 adolescents 59.1 % of adolescents were classified as normal weight, 15.2 % as overweight and 25.7 % as obese. Pediatric HRQoL scores across the total and the four specific domains reported by parents were lower than by adolescents. For parent proxy-reports, the mean scores of physical, emotional, social, and school functioning were 72.7, 73.3, 73.3, and 68.0, respectively. For adolescent self-reports, the mean scores of physical, emotional, social, and school functioning were 82.4, 79.0, 85.0, and 72.6, respectively. ICC analyses show a moderate agreement between pediatric HRQoL rated by adolescents and parents: ICCs = 0.63 for total HRQoL, 0.51 for physical functioning, 0.40 for emotional functioning, 0.50 for social functioning, 0.50 for school functioning, and 0.50 for total functioning.

**Table 1:** Sample characteristics (*n* = 323)

Variables	Mean (SD*)	Range
Adolescent characteristics		
Age	17.1 (1.0)	14–19
Gender (female)	51.7 %	-
Race		
White	41.5 %	-
Black	37.5 %	-
Hispanics	12.4 %	-
Others	8. 7 %	-
Body mass index		
Normal weight (BMI ≥5th and <85th percentile)	59.1 %	-
Overweight (BMI > = 85th and <95th percentile)	15.2 %	-
Obese (BMI > = 95th percentile)	25.7 %	-
Total functioning		
Adolescent self-reports	80.1 (12.7)	28–100
Parent proxy-reports	71.8 (15.8)	25–100
Physical functioning		
Adolescent self-reports	82.4 (17.3)	20–100
Parent proxy-reports	72.7 (21.4)	20–100
Emotional functioning		
Adolescent self-reports	79.0 (18.3)	20–100
Parent proxy-reports	73.3 (18.5)	20–100
Social functioning		
Adolescent self-reports	85.0 (16.6)	20–100
Parent proxy-reports	73.3 (20.3)	20–100
School functioning		
Adolescent self-reports	72.6 (15.3)	20–100
Parent proxy-reports	68.0 (18.1)	20–100
Parent characteristics		
Age	39.9 (11.9)	20–83
Gender (female)	86 %	-
Race		
White	42.7 %	-
Black	36.2 %	-
Hispanics	16.1 %	-
Others	5.0 %	-
Education		
Below high school	26.3 %	-
High school	35.6 %	-
Some college or associate degree	30.0 %	-
Undergraduate/bachelor degree or above	8.1 %	-

*SD standard deviation

### Differences in pediatric HRQoL rated by adolescents (and parents) across BMI weight categories

Table 2 shows that given a particular BMI weight category, the adolescent rating of HRQoL (unit: mean (SD)) were consistently higher in all domains of HRQoL than the parent’s rating (*p*’s <0.05) except school functioning for overweight adolescents. The magnitude of differences was above 4.5 points (defined as clinically meaningful differences [30]) in all domains for a particular BMI weight category, except emotional and social functioning for normal weight adolescents (3.4 and 3.3, respectively). Interestingly, the difference within the dyadic ratings of HRQoL increased alongside the increase in BMI severity. Regardless of the specific domains, a greater discrepancy in the dyadic ratings of pediatric HRQoL was observed for adolescents with excess body weight compared to adolescents with normal weight. For example, the effect size of the total HRQoL for adolescents with excess body weight was 0.6, which was larger than the effect size 0.4 for adolescents with normal weight.

**Table 2:** Comparison of adolescent self-reports and parent proxy-reports of total and domain-specific HRQoL for each BMI weight category^a^

	Adolescent self-reports: mean (SD)					Parent proxy-reports: mean (SD)					Difference between adolescent self-reports and parent proxy-reports: mean (SD)				
	PF	EF	SoF	ScF	Total	PF	EF	SoF	ScF	Total	PF	EF	SoF	ScF	Total
Normal weight	82.7 (17.7)	78.1 (18.3)	83.8 (18.4)	72.5 (15.0)	79.0 (13.2)	74.1 (20.8)	74.7 (18.7)	73.9 (20.7)	69.2 (16.7)	72.9 (15.5)	8.6* (0.5)	3.4* (0.2)	9.9* (0.5)	3.3* (0.2)	6.1* (0.4)
Overweight	83.0 (18.0)	80.7 (15.5)	86.7 (13.6)	73.6 (13.6)	81.8 (9.9)	71.5 (23.9)	70.7 (17.8)	71.6 (21.2)	68.9 (20.8)	70.9 (16.3)	11.5* (0.5)	10.0* (0.5)	15.1* (0.6)	4.7 (0.2)	10.9* (0.6)
Obese	81.8 (17.5)	79.1 (20.1)	86.8 (13.9)	72.8 (16.1)	80.3 (13.3)	69.4 (21.4)	71.4 (19.1)	74.0 (18.7)	66.1 (19.4)	70.6 (16.5)	12.4* (0.6)	7.7* (0.4)	12.8* (0.6)	6.7* (0.4)	9.7* (0.6)
Obese & Overweight	82.2 (17.6)	79.7 (18.5)	86.8 (13.7)	73.1 (15.2)	80.8 (12.1)	70.1 (22.2)	71.2 (18.5)	73.1 (19.6)	67.1 (19.9)	70.7 (16.3)	12.1* (0.5)	8.5* (0.4)	13.7* (0.6)	6.0* (0.3)	10.2* (0.6)

*BMI* body mass index, *HRQoL* health-related quality of life, *PF* physical functioning, *EF* emotional functioning, *SoF* social functioning, *ScF* school functioning

*p < 0.05

^a^Analyses were based on Wilcoxon signed rank tests

### Multiple regression analysis with robust standard errors

Table 3 shows the factors associated with the difference in pediatric HRQoL rated by adolescents and parents using multiple linear regression analyses. Factors under investigation included the adolescent’s age, gender, BMI weight categories, and parent’s race and educational background. Results suggest that excess body weight was associated with a greater discrepancy in the dyadic ratings of total and emotional functioning (*p*’s <0.05) when controlling for the other demographic characteristics of the dyads. In addition, older adolescents were associated with greater discrepancies in the dyadic rating of the emotional functioning domain (*p* < 0.05). None other demographic characteristics had a significant association with the differences in the dyad scores.

**Table 3:** Differences in adolescent self-reports and parent proxy-reports of total and domain-specific HRQoL associated with weight status after adjusting for covariates^a^

Difference in HRQoL rated by adolescent self-reports and parent proxy- reports	Physical functioning	Emotional functioning	Social functioning	School functioning	Total
	Unstandardized coefficient (S.E.)				
Covariate					
Age	1.03 (1.11)	2.88* (1.15)	0.49 (1.14)	−0.02 (1.20)	0.87 (1.03)
Sex	0.62 (2.14)	−0.31 (2.31)	0.41 (2.36)	3.22 (2.18)	0.98 (1.93)
Education^b^					
Below high school	2.38 (2.82)	5.21 (2.77)	0.74 (3.05)	−1.24 (3.17)	2.40 (2.67)
Some college or associate degree	2.16 (2.48)	0.50 (2.97)	−2.73 (3.06)	2.84 (2.89)	1.28 (2.54)
Undergraduate/bachelor’s degree	0.25 (4.28)	5.30 (5.22)	6.10 (3.64)	4.27 (4.37)	4.49 (3.75)
Parent’s race^c^					
Black	2.98 (2.44)	−2.85 (2.61)	−0.58 (2.71)	−0.76 (2.75)	−0.37 (2.34)
Hispanic	1.85 (3.13)	1.96 (3.65)	0.53 (3.22)	0.31 (3.18)	0.87 (2.80)
Others	−3.25 (3.82)	−1.91 (4.42)	−7.95 (6.16)	−1.17 (4.63)	−2.82 (4.36)
Overweight & obesity^d^	2.90 (2.32)	5.40* (2.77)	4.35 (2.43)	3.64 (2.39)	4.46* (2.06)

*S.E.* standard error

*p < 0.05

^a^Analyses were based on multiple linear regression with robust standard error estimation

Reference group: ^b^high school; ^c^White; ^d^normal weight category

### Difference in pediatric HRQoL between BMI weight categories based on adolescent self-reports and parent proxy-reports

Table 4 shows the difference in the ratings of pediatric HRQoL across different BMI weight categories. Generally, parents reported that adolescents with normal weight had greater total and domain-specific HRQoL than adolescents with excess body weight or obesity alone. However, the differences in HRQoL between different BMI weight categories in adolescents were all less than the meaningful cutoff (4.5 points [30]) and not statistically significant (*p* > 0.05). The only exception was the differences in physical functioning between normal weight and obese adolescents, which was greater than five points but not statistically significant (*p* > 0.05). For adolescent self-reports, the total HRQoL as well as the emotional and social functioning were greater in overweight and obese adolescents than normal weight adolescents. However, the difference was not statistically significant (*p* > 0.05).

**Table 4:** Adolescent self-reports and parent proxy-reports of total and domain-specific HRQoL by BMI weight categories^a^

	HRQoL scores by BMI weight categories: mean (SD)				Differences between BMI weight categories: mean (SD)			
	Normal weight	Overweight	Obese	Overweight & obese	Overweight vs. normal weight	Obese vs. normal weight	Obese vs. overweight	Overweight & obese vs. normal weight
Adolescent self-reports								
Total	79.8 (13.1)	80.8 (10.4)	80.3 (12.9)	80.5 (12.1)	1.0 (0.0)	0.5 (0.0)	−0.5 (−0.0)	0.7 (0.0)
Physical functioning	82.7 (17.0)	82.3 (18.1)	81.6 (17.7)	81.9 (17.8)	−0.4 (−0.0)	−1.1 (−0.0)	−0.8 (−0.0)	−0.8 (−0.0)
Emotional functioning	78.5 (18.1)	80.7 (15.5)	79.2 (20.0)	79.8 (18.5)	2.2 (0.0)	0.7 (0.0)	−1.5 (−0.0)	1.2 (0.0)
Social functioning	84.0 (18.2)	86.5 (13.5)	86.4 (14.0)	86.4 (13.8)	2.5 (0.0)	2.4 (0.0)	−0.1 (−0.0)	2.5 (0.0)
School functioning	72.6 (15.4)	72.6 (14.1)	72.4 (15.9)	72.4 (15.2)	−0.1 (−0.0)	−0.3 (−0.0)	−0.2 (−0.0)	−0.2 (−0.0)
Parent proxy-reports								
Total	72.5 (15.6)	71.9 (15.9)	70.1 (16.3)	70.8 (16.1)	−0.6 (−0.0)	−2.4 (−0.1)	−1.7 (−0.1)	−1.7 (−0.1)
Physical functioning	74.5 (20.6)	72.0 (23.4)	69.1 (21.5)	70.1 (22.2)	−2.6 (−0.0)	−5.4 (−0.1)	−2.9 (−0.1)	−4.4 (−0.1)
Emotional functioning	74.8 (18.5)	71.0 (17.7)	71.5 (19.0)	71.3 (18.4)	−3.8 (−0.1)	−3.3 (−0.1)	0.5 (0.0)	−3.4 (−0.1)
Social functioning	73.7 (20.9)	71.8 (21.1)	73.3 (18.7)	72.8 (19.5)	−1.9 (−0.0)	−0.4 (−0.0)	1.5 (0.0)	−0.9 (−0.0)
School functioning	68.4 (17.0)	69.9 (20.0)	66.0 (19.3)	67.4 (19.6)	1.5 (0.1)	−2.4 (−0.1)	−3.9 (−0.1)	−0.9 (−0.0)

^a^Analyses were based on Mann–Whitney U-tests

### DIF associated with BMI weight categories by adolescent self-reports and parent proxy-reports

Table 5 shows the results of DIF analysis associated with BMI weight categories. If a specific item was identified with DIF, the item parameters are reported. Overall, adolescent self-reports had relatively fewer DIF items than parent proxy-reports (two for adolescent self-reports and five for parent proxy-reports). For adolescent self-reports, when comparing excess body weight or obese adolescents to normal weight adolescents, items #9 (feeling afraid/scared) and #10 (feeling sad/blue) of emotional functioning were flagged with DIF. When comparing obese adolescents to overweight adolescents, items #9 were flagged with DIF. However, the directions on item #9 and #10 were different. For excess body weight vs. normal weight, conditioning on the same underlying emotional functioning, excess body weight adolescents reported greater mean scores or better HRQoL for items #9 (0.2 units higher), but lower mean scores for and #10 (0.2 units lower) than did normal weight adolescents.

**Table 5:** DIF identification and parameter estimates for specific items measuring HRQoL for adolescent self-reports and parent proxy-reports by weight categories

	Domains and DIF items	Estimate	Standard error	*P*-value
Adolescent self-reports				
Overweight & obese vs. normal weight (reference group)				
Emotional functioning				
Item 9 (feeling afraid/scared)		0.18	0.09	0.046
Item 10 (feeling sad/blue)		−0.20	0.10	0.046
Obese vs. normal weight (reference group)				
Emotional functioning				
Item 9 (feeling afraid/scared)		0.28	0.10	0.007
Item 10 (feeling sad/blue)		−0.27	0.12	0.020
Obese vs. overweight (reference group)				
Emotional functioning				
Item 9 (feeling afraid/scared)		0.30	0.14	0.031
Parent proxy-reports				
Overweight & obese vs. normal weight (reference group)				
Emotional functioning				
Item 9 (feeling afraid/scared)		−0.28	0.12	0.015
Social functioning				
Item 15 (other teens not wanting to be his/her friend)		−0.28	0.12	0.019
Overweight vs. normal weight (reference group)				
Emotional functioning				
Item 11 (feeling angry)		0.35	0.16	0.031
Social functioning				
Item 16 (getting teased by other teens)		0.39	0.19	0.039
Obese vs. normal weight (reference group)				
Physical functioning				
Item 4 (lifting something heavy)		−0.30	0.15	0.047
Emotional functioning				
Item 9 (feeling afraid/scared)		−0.35	0.13	0.007
Obese vs. overweight (reference)				
Social functioning				
Item 16 (getting teased by other teens)		−0.45	0.19	0.011

For parent proxy-reports, when comparing adolescents with excess body weight to adolescents with normal weight, #9 of emotional functioning and #15 (other teens not wanting to be his/her friend) of social functioning was flagged with DIF. Conditioning on the same underlying emotional functioning, parents of adolescents with excess body weight reported lower score for item #9 and #15 (0.3 and 0.3 units, respectively) than parents of adolescents with normal weight. When comparing overweight to normal weight adolescents, #11 (feeling angry) of emotional functioning and #16 (getting teased by other children) of social functioning were flagged with DIF. Conditioning on the same underlying functioning, parents of overweight adolescents reported higher scores for items #11 and #16 (0.4 and 0.4 units, respectively) than parents of normal weight adolescents. When comparing obese to normal weight adolescents, items #4 (lifting something heavy) in physical functioning and item #9 in emotional functioning were flagged with DIF. Conditioning on the same underlying functioning, parents of obese adolescents reported lower scores for item #4 and item #9 (0.3 and 0.4 units, respectively) than parents of overweight adolescents. Finally, when comparing obese to overweight adolescents, items #16 was flagged with DIF. Conditioning on the same underlying functioning, parents of obese adolescents reported lower scores (0.5 units) than parents of overweight adolescents.

## Discussion

HRQoL is an important outcomes indicator for managing people with chronic conditions, such as obesity. Though studies have evaluated the relationship between excess body weight and HRQoL in pediatric populations, few studies have taken the measurement properties into consideration. In this study, we found that given a particular weight category, HRQoL ratings by adolescents themselves were significantly higher than their parent proxy-reports across all domains, except for school functioning among overweight adolescents. Extending previous work [3, 31, 32], we specifically found that BMI weight categories in adolescents were associated with a difference in the rating of pediatric HRQoL by adolescents and their parents. The difference in the dyadic ratings increased as the BMI severity increased, which was confirmed by multivariable analyses where excess body weight was associated with a greater discrepancy in the dyadic rating in the total HRQoL and emotional functioning (Table 3).

Greater impairment in HRQoL reported by parents than by adolescents across different BMI weight categories in adolescents was in line with previous pediatric literature [6–8, 10–12] suggesting that parents might possess limited understanding of their children’s life experiences associated with excess body weight. Additionally, overweight and obese adolescents might communicate less often with their parents about how they feel and think [11]; in combination with parents’ feelings of helplessness, guilt, and negative evaluations of their adolescent’s health status, this lack of communication may contribute to the difference in the dyadic ratings. It is also possible that excess body weight experienced by the family or parents themselves may lead to using a higher standard to rate their adolescent’s HRQoL. Obesity clustering within families is a common phenomenon, as Lake and colleagues reported that obese adolescents lived with obese family members [33]. Our results echo previous studies with respect to the use of both parent proxy-reports and adolescent self-reports for assessing adolescents’ HRQoL in interventional studies [6, 7]. Given that body weight can affect the estimation of adolescent HRQoL rated by adolescents and parents, it is important to collect HRQoL data from both parents and adolescents especially when the studies involve adolescents with a wide range of body weights.

We compared pediatric HRQoL across different BMI weight categories in adolescents and found that the differences were not significant across these categories either by adolescent self-reports or parent proxy-reports. This finding is in line with several previous studies [11, 12], but contradict other studies that had observed significantly impaired HRQoL in obese adolescents compared to their lean counterparts using both self-reports and proxy-reports [8, 9, 34]. The reasons behind the non-discernible difference in pediatric HRQoL across different weight categories in adolescents are complex and may be confounded by psychosocial factors such as social desirability and weight-related stigma or discrimination. Evidence suggests that social desirability, either intentional or self-deceptive [35], may drive underreporting of obesity-related measures such as body weight status and dietary consumption [36, 37]. The same psychological mechanism may link the excess body weight to the over reporting of HRQoL. In contrast, one can argue that people with excess body weight might experience weight-related stigma and social discrimination, which in turn leads to poor HRQoL [38–40]. Indeed, obese children are likely to experience increased teasing and bullying behavior and lower levels of involvement in social activities; peers and educators may hold biased views of children with excess body weight, which may relate to weight stigmatization [5]. However, studies have found that the negative relationship between stigma and HRQoL is mediated by internalized societal attitude or perceived discrimination [41], and African-American women seem to have more positive attitudes toward obesity than White women and men [42]. Because our study focuses on Medicaid enrollees which comprised of a greater proportion of African-Americans (38 %) than that of the national average (15 %) [43], we may include more individuals with positive attitudes toward obesity, leading to non-discernible differences in adolescent HRQoL across different weight categories.

DIF test serves an empirical methodology to demonstrate measurement bias in the rating of HRQoL items across BMI weight status. This study identified two DIF items in the adolescent self-reports and five DIF items in the parent-proxy reports. We found that parents are more likely to use different perspectives to interpret DIF items per specific weight category compared to adolescents themselves. For example, the item “afraid/scared” (item #9) was flagged as DIF by both adolescent self-reports and parent proxy-reports . However, the direction of DIF for this item was opposite between adolescent self-reports and parent proxy-reports. When comparing excess body weight/obese adolescents to normal weight adolescents or comparing obese adolescents to overweight adolescents, those adolescents with heavier/unhealthier body weight reported less afraid or scared than those with healthier body weight. However, the result derived from adolescent self-reports was in contrast to the parent proxy-reports. Although we cannot ascertain the potential sources of DIF in this study, we suspect that, in contrast to adolescents themselves, parents of obese adolescents might have more obesity-relevant concerns, leading to a negative attitude towards society’s perception and stigmatization of their adolescent’s excess body weight [5]. Compared to adolescents, parents may have more experience and understanding regarding the harmful effect of obesity in daily activities of adolescents [7]. The DIF findings may also reflect the differential adjustment by parents and adolescents for adolescents’ HRQoL associated with excess body weight. Interestingly, the directions of DIF items were inconsistent even if they were within the same HRQoL domain. For example, albeit items #9 and #10 capturing emotional functioning, excess body weight adolescents reported less afraid/scared (items #9), but more sad/blue (#10) than did normal weight adolescents. This implies that adolescents with different body weights may possess the opposite meanings for the phrases “afraid/scared” and “sad/blue” because of different experiences with being afraid/scared or being sad/blue. Understanding how parents and adolescents perceive adolescent’s HRQoL may assist researchers in reducing discrepancies when interpreting HRQoL items. Further research is encouraged to use cognitive interviewing techniques to understand the psychological mechanisms behind the DIF findings [19].

Several limitations should be noted. First, this study was limited to adolescents from low-income families enrolled in the Florida KidCare program, which may limit the external validity of these findings to general populations. Nevertheless, it is imperative to focus on low-income families because they are at a higher risk of developing obesity [19]. Second, this study did not collect and control for parents’ body weight status, psychological status (e.g., depression), and their HRQoL. It is plausible that parents’ body weight status, depression, and HRQoL may bias their observations of their adolescent’s HRQoL. Third, adolescents’ height and weight were reported by the parents rather than direct measures, which highly related to parents’ psychological status and HRQoL and may potentially lead to a misclassification of body weight and severity. Nevertheless, recent evidence suggests that a parental report is a better indicator of obesity than an adolescent’s self-report [44]. Fourth, the cross-sectional design of our study makes it improper to infer a causal link between excess body weight and HRQoL [3]. An individual’s perceptions of his/her own HRQoL and physical appearance might influence his/her lifestyle, which in turn may affect body weight [3]. Finally, the data available for this study did not include some variables that potentially confound the association between excess body weight and HRQoL, such as eating habits, self-esteem, duration of physical activity, and interpersonal behavior [3, 5].

## Conclusion

In summary, excess body weight was associated with a difference in pediatric HRQoL rated by adolescents and their parents. However, pediatric HRQoL did not vary significantly across different BMI categories by adolescent self-reports or parent proxy-reports. More DIF (or bias response) in PedsQL associated with BMI weight categories in adolescents was identified in parent proxy-reports than adolescent self-reports. DIF assessment is useful for investigating the measurement equivalence and adjustment for the rating of HRQoL associated with BMI weight categories.
